# Using Detergent to Enhance Detection Sensitivity of African Trypanosomes in Human CSF and Blood by Loop-Mediated Isothermal Amplification (LAMP)

**DOI:** 10.1371/journal.pntd.0001249

**Published:** 2011-08-02

**Authors:** Dennis J. Grab, Olga V. Nikolskaia, Noboru Inoue, Oriel M. M. Thekisoe, Liam J. Morrison, Wendy Gibson, J. Stephen Dumler

**Affiliations:** 1 Department of Pathology, The Johns Hopkins University School of Medicine, Baltimore, Maryland, United States of America; 2 National Research Center for Protozoan Diseases, Obihiro University of Agriculture and Veterinary Medicine, Obihiro, Japan; 3 Department of Zoology and Entomology, University of the Free State, Qwaqwa Campus, Phuthaditjhaba, South Africa; 4 Wellcome Trust Centre for Molecular Parasitology, University of Glasgow, Glasgow, United Kingdom; 5 School of Biological Sciences, University of Bristol, Bristol, United Kingdom; New York University School of Medicine, United States of America

## Abstract

**Background:**

The loop-mediated isothermal amplification (LAMP) assay, with its advantages of simplicity, rapidity and cost effectiveness, has evolved as one of the most sensitive and specific methods for the detection of a broad range of pathogenic microorganisms including African trypanosomes. While many LAMP-based assays are sufficiently sensitive to detect DNA well below the amount present in a single parasite, the detection limit of the assay is restricted by the number of parasites present in the volume of sample assayed; i.e. 1 per µL or 10^3^ per mL. We hypothesized that clinical sensitivities that mimic analytical limits based on parasite DNA could be approached or even obtained by simply adding detergent to the samples prior to LAMP assay.

**Methodology/Principal Findings:**

For proof of principle we used two different LAMP assays capable of detecting 0.1 fg genomic DNA (0.001 parasite). The assay was tested on dilution series of intact bloodstream form *Trypanosoma brucei rhodesiense* in human cerebrospinal fluid (CSF) or blood with or without the addition of the detergent Triton X-100 and 60 min incubation at ambient temperature. With human CSF and in the absence of detergent, the LAMP detection limit for live intact parasites using 1 µL of CSF as the source of template was at best 10^3^ parasites/mL. Remarkably, detergent enhanced LAMP assay reaches sensitivity about 100 to 1000-fold lower; i.e. 10 to 1 parasite/mL. Similar detergent-mediated increases in LAMP assay analytical sensitivity were also found using DNA extracted from filter paper cards containing blood pretreated with detergent before card spotting or blood samples spotted on detergent pretreated cards.

**Conclusions/Significance:**

This simple procedure for the enhanced detection of live African trypanosomes in biological fluids by LAMP paves the way for the adaptation of LAMP for the economical and sensitive diagnosis of other protozoan parasites and microorganisms that cause diseases that plague the developing world.

## Author Summary

Human African trypanosomiasis or sleeping sickness is a fatal disease (if untreated) spread by bloodsucking tsetse flies. Trypanosome parasites first enter the blood and lymph and eventually invade the brain. In rural clinical settings, diagnosis still relies on the detection of these microbes in blood and cerebrospinal fluid (CSF) by microscopy. LAMP, or loop-mediated isothermal amplification of DNA, is a technique that can specifically detect very small amounts of DNA from an organism. It is similar to PCR, the polymerase chain reaction, another DNA amplification technique widely used for diagnosis of infectious diseases. LAMP's advantages are that the reaction works at one temperature, whereas PCR needs a thermocycler, and LAMP is not affected by blood components that inhibit PCR. We show that by simply adding detergent during sample preparation, the analytical sensitivity of LAMP targeting many gene copies is greatly improved, presumably because DNA is released from the pathogen cells and dispersed through the sample. To demonstrate proof of principle, we used pathogenic trypanosomes in different human body fluids (CSF or blood), but this simple modification should be applicable for diagnosis of other microbial infections where cells are sensitive to detergent lysis.

## Introduction

Tsetse fly-transmitted African trypanosomes are major pathogens of humans and livestock. Two subspecies of *Trypanosoma brucei* (*T. b. rhodesiense* and *T. b. gambiense*) cause human African trypanosomiasis (HAT, commonly called sleeping sickness). After replicating at the tsetse fly bite site, trypanosomes enter the hemolymphatic system (early stage or stage 1) (5, 9). Without treatment, the parasites go on to invade the central nervous system (CNS; late stage or stage 2), a process that takes months to years with *T. b. gambiense* (West and Central African HAT) or weeks to months with *T. b. rhodesiense* (East African HAT). The parasites cause a meningoencephalitis leading to progressive neurologic involvement with concomitant psychiatric disorders, fragmentation of the circadian sleep-wake cycle and ultimately to death if untreated (4, 5, 9).

A key issue in the treatment of HAT is to distinguish stage 1 from stage 2 disease, as the drugs used for the treatment of stage 2 need to cross the blood-brain barrier [1], [2]. The most widely used drug is melarsoprol (developed in 1949), which is effective for *T. b. gambiense* and *T. b. rhodesiense* HAT, but unfortunately, melarsoprol leads to severe and fatal encephalitis in about 5–10% of recipients despite treatment for this condition [3], [4], [5]. Therefore, where HAT is endemic, accurate staging is critical, because failure to treat CNS involvement leads to death, yet inappropriate CNS treatment exposes an early-stage patient unnecessarily to highly toxic and life-threatening drugs.

The diagnosis of HAT in the rural clinical setting, where most patients are found, still relies largely on the detection of parasitemia by blood smear and/or CSF microscopy [6], [7]. While *T. b. rhodesiense* detection in blood is frequently successful, *T. b. gambiense* infections, which constitute over 90% of all HAT cases, typically show very low parasitemias, and concentration techniques such as centrifugation or mini-anion exchange columns are usually necessary [6], [7], [8]. Stage determination still relies on lumbar puncture to examine CSF for trypanosomes or white cell count/protein concentration suggestive of chronic meningoencephalitis. Threshold values for these parameters are controversial, with the conventional value for stage 2 (>5 cells/µL) now increased to >10 or even 20 cells/µL [9]. In summary, diagnosis and staging of HAT is currently time consuming, intensive and difficult.

DNA-based diagnostic methods such as PCR and LAMP now offer greater sensitivity than existing diagnostic methods, detecting DNA from the equivalent of 0.01 parasites or less. Based on PCR protocols for HAT [10], we described LAMP targeting the conserved paraflagellar rod A (PRFA) gene in all *T. brucei* subspecies and *T. evansi* [11]. LAMP is an isothermal DNA amplification method with excellent analytical sensitivity and specificity when employed for the detection of a variety of microorganisms (reviewed in [12]), including human and animal infective African trypanosomes [11], [13], [14], [15], [16], [17], [18], [19], [20], [21]. LAMP relies on autocycling strand displacement coupled to DNA synthesis by *Bst* DNA polymerase, a reaction similar to rolling-circle amplification [22] but with the added advantage that a heat-denaturing step is not necessary to initiate rounds of amplification. Specificity is dictated by four primers (F3, B3, FIP and BIP), and the addition of two loop primers (LoopF and LoopB) significantly reduces the reaction time [23].

LAMP is cost-effective (<1 US dollar/test), simple (the isothermal reaction requires a simple heating device), and rapid (within 60 minutes) [12], [24]. Furthermore, *Bst* DNA polymerase can be stored for weeks at ambient temperatures, a critical property where maintaining a cold chain is difficult [13]. Positive reactions are indicated by turbidity [25], color changes after addition of hydroxynaphthol blue (HNB) [26], or changes in fluorescence using indicator dyes [26], [27], [28].

Despite its advantages, the usefulness of LAMP for HAT diagnosis is handicapped in the clinical setting by its inability to directly detect live trypanosomes in blood or CSF below 1 parasite/µL (10^3^ parasites/mL), the practical detection limit based on a 1 µL sample volume often used in LAMP or PCR assays. While sensitivity can be increased 5–10 fold by adding more sample volume, significant improvement in the assay system for the detection of live parasites in biologically relevant samples would clearly be of benefit for diagnosis. Here, we introduce a very simple modification to the LAMP assay recognizing multi-copy gene targets that can increase the analytical sensitivity for the detection of live parasites 100-fold or more.

## Materials and Methods

### LAMP

**Figure 1:**
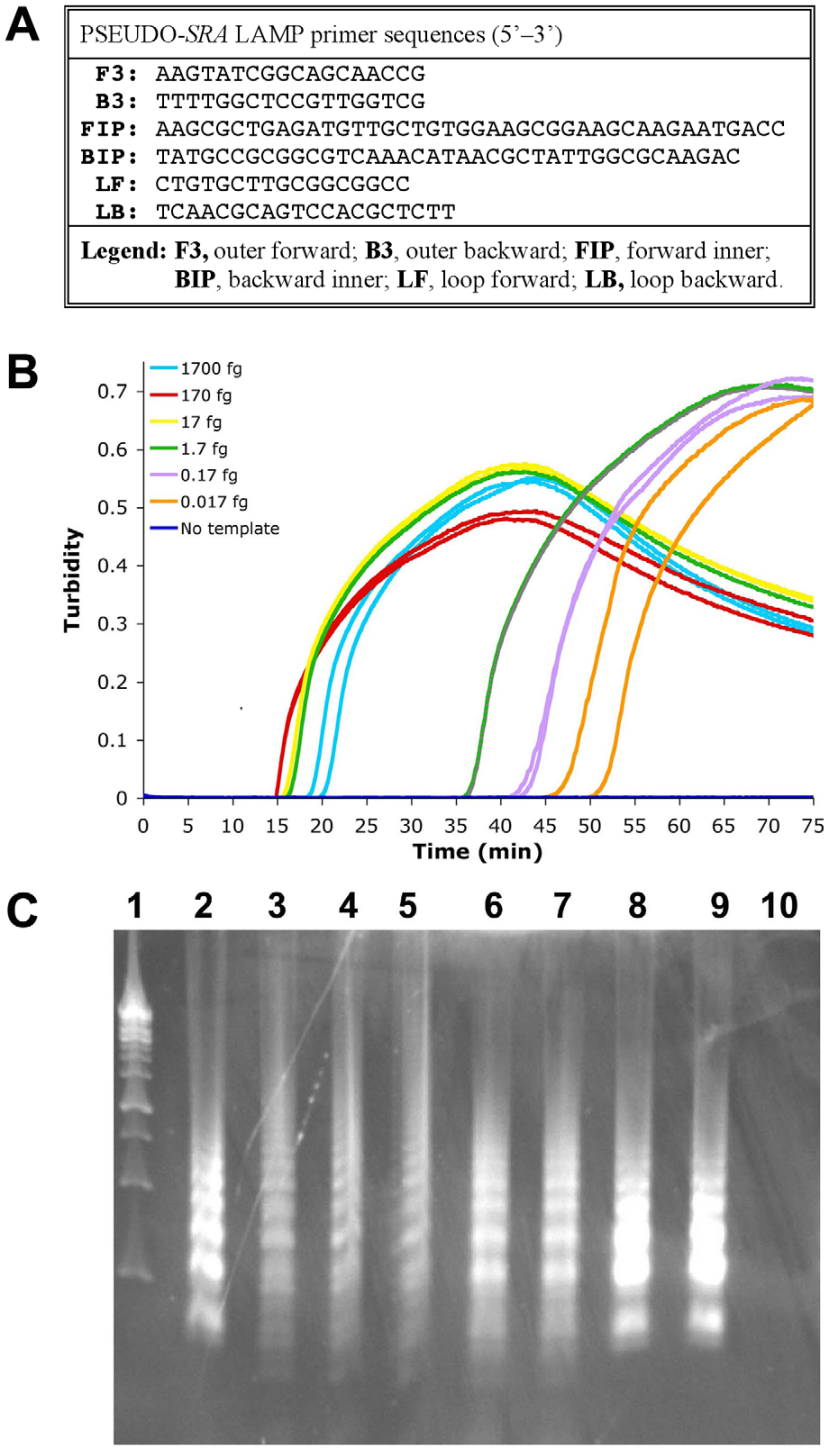
PSEUDO-*SRA* LAMP for the detection of *T. b. rhodesiense* genomic DNA. The PSEUDO-*SRA* LAMP primer set (Panel A) was tested with 1∶10 serially diluted *T. b. rhodesiense* IL1852 DNA (1700 fg to 0.017 fg) (Panel B). Replicates: Samples with DNA, n = 2; without DNA, n = 4. The data for each individual sample is presented as real-time turbidity values versus LAMP reaction time. As shown in Panel C, 5 µL reaction product after PSEUDO-*SRA* LAMP or *SRA* LAMP amplification of differing concentrations of *T. b. rhodesiense* IL1852 template were electrophoresed through 2% agarose gel containing ethidium bromide. From left to right: **Lane 1**, 1 kb DNA ladder (Fermentas); **Lane 2**, 17 pg IL 1852 DNA after *SRA* LAMP (Njiru [18]). **Lane 3**, 17 pg IL 1852 DNA after PSEUDO-*SRA* LAMP. Lanes 4–10, show a dilution series of template IL1852 DNA as follows: **Lane 4**, 1700 fg; **Lane 5**, 170 fg; **Lane 6**,17 fg; **Lane 7**, 1.7 fg; **Lane 8**, 0.17 fg; **Lane 9**, 0.017 fg; **Lane 10**, no DNA template.

Two LAMP primer sets were tested. Data on analytical sensitivity and specificity of a LAMP primer set for trypanosome DNA based on the multicopy (approximately 500 copies) repetitive insertion mobile element (RIME) of subgenus *Trypanozoon* (GenBank Accession No. K01801) is well-documented [16], [26], [29]. Using between 2–4 µL sample, this pan-*T. brucei* assay is reported to detect DNA from ∼0.001 trypanosome [16]. LAMP primers based on the serum resistance associated (*SRA*) gene (GenBank AJ560644) (see Table S1 for gene sequence) were designed using PrimerExplorer version 4 software (http://primerexplorer.jp/e/) to create the basic F3, B3, FIP, BIP [30] and loop LF, LB [23] primers (Fig. 1A). As this assay recognized more than the *SRA* gene, this primer set is designated PSEUDO-*SRA*. All RIME and PSEUDO-*SRA* LAMP primers were synthesized and HPLC purified. For comparison, we also used the *SRA* gene (GenBank accession number Z37159)-specific LAMP assay [17]. Genomic DNA was prepared by using Qiagen DNeasy Blood & Tissue Kits.

The LAMP reaction was performed as previously described [11], [14], [15]. Briefly, the reaction contained 12.5 µL of 2x LAMP buffer (40 mM Tris-HCl [pH 8.8], 20 mM KCl, 16 mM MgSO_4_, 20 mM [NH_4_]_2_SO_4_, 0.2% Tween 20, 1.6 M Betaine, 2.8 mM of each deoxyribonucleotide triphosphate), 1.0 µL primer mix (5 pmol each of F3 and B3, 40 pmol each of FIP and BIP) or 1.3 µL when LF and LB (20 pmol each) were included, 1 µL (8 units) *Bst* DNA polymerase (New England Biolabs, Tokyo, Japan), 1 µL of template DNA. Final volumes were adjusted to 25 µL with distilled water. All reactions were conducted in 2 to 4 replicates and were monitored in real-time in a Loopamp® real-time turbidimeter LA320C (Teramecs, Tokyo, Japan). The optimal reaction temperatures were 62°C (RIME LAMP) and 63°C (PSEUDO-*SRA* LAMP). The reaction was terminated by increasing the temperature to 80°C for 5 min. In addition to turbidity, the amplified products were analyzed on 2% agarose gels using the E-Gel EX system with ethidium bromide or SYBR green incorporated into the gels (Invitrogen), and/or after addition of hydroxynaphthol blue (HNB) [26] to enable visual detection. The HNB color change from violet to sky blue has been consistently interpreted by independent observers as the easiest to see [26].

### Analytical sensitivity using human CSF spiked with trypanosomes

Human CSF was obtained as discarded samples from The Johns Hopkins Hospital Microbiology laboratory with approval of the Johns Hopkins Medicine IRB. CSF were adjusted to contain either 1/20 volume deionized water (untreated CSF) or 1/20 volume 10% (w/v) Triton X-100 (final concentration 0.5% Triton). A 10% (w/v) Triton X-100 stock solution was made by adding 1 g Triton X-100 to a final volume of 10 mL DNase/RNase free water (Qiagen). We used bloodstream form *T. b. rhodesiense* IL1852, a CSF isolate from a patient in Kenya [31], [32]. Originally thought to be *T. b. gambiense* it has been reclassified as *T. b. rhodesiense* [33] based on the presence of the *SRA* gene [34] and the absence of the *TgsGP* gene [35], [36] (Fig. S1). Human CSF was spiked with bloodstream form *T. b. rhodesiense* IL1852 and the samples serially diluted 1∶10 in CSF with or without 0.5% detergent to cover a range of parasite concentrations from 10^4^ to 10^−1^ parasites/mL. After 60 min incubation at ambient temperature to allow for lysis, the LAMP assays were done using 1 µL CSF.

### Analytical sensitivity using human blood spiked with trypanosomes

In the field, biological samples are often shipped to another geographical site for later analyses. They are often preserved by spotting on paper cards designed for short-term protein, RNA and DNA storage (2 weeks at ambient temperature) such as Whatman Protein Saver 903, or long-term (years) DNA storage/archiving on Whatman FTA cards. To simulate these conditions, human blood obtained as discarded samples from The Johns Hopkins Hospital Microbiology laboratory with approval of the Johns Hopkins Medicine IRB was spiked with *T. b. rhodesiense* (10^4^ to 10^−2^ parasites/mL). Protein Saver 903 cards were pretreated with 50 µL 0.5% Triton X-100, which was sufficient to fill the designated circle on the cards, and dried overnight prior to whole blood spotting. For assay standardization, three 2 mm punches made from the 1 cm^2^ dried blood spots (DBS) and DNA was extracted using standard methods [37]. The LAMP assays were done using 1 µL DBS DNA template. Alternatively, untreated and 0.5% Triton X-100 treated trypanosome-spiked blood were spotted on untreated Protein Saver 903 cards and dried overnight with subsequent DBS DNA extraction as above.

## Results and Discussion

### LAMP with genomic parasite DNA

Based on experiments repeated at least 3 times, LAMP assays successfully amplified *T. b. rhodesiense* DNA within 55–60 min at 62°C (RIME LAMP) or 63°C (PSEUDO-*SRA* LAMP). As reported previously [16], we found that RIME LAMP detected 0.1 fg genomic DNA (0.001 parasite) from *T. b. rhodesiense* IL1852 (not shown). PSEUDO-*SRA* LAMP was as sensitive and reliably detected 0.1 fg (0.001 parasite) or less *T. b. rhodesiense* IL1852 genomic DNA (Fig. 1B and 1C). Nonetheless, when using the *SRA* gene specific LAMP assay [17] the detection limit for *T. b. rhodesiense* IL1852 genomic DNA was 0.1–1.0 pg (1–10 parasites), comparing favorably to reported values [17].

The standard curves with PSEUDO-*SRA* LAMP seem to display biphasic kinetics with an early initial phase (15–20 min) followed by a late second phase (35 and 55 min) with a break point around 1.7 fg DNA (Fig. 1B) suggesting that it targets other genomic components besides the *SRA* gene. As *SRA* is a truncated VSG, it is likely that the PSEUDO-*SRA* LAMP is amplifying other *VSG* sequences, albeit not efficiently (see below). Although the PSEUDO-*SRA* LAMP primer sequences were verified as being unique by BLAST analysis of the *T. b. brucei* TREU 927 genome sequence and the VSG database (TriTrypDB: http://tritrypdb.org/tritrypdb/), *VSG* repertoires are diverse between strains, and we were unable to assess the primers against sequences of the full IL1852 *VSG* repertoire as its genome has not been sequenced.

The PSEUDO-*SRA* LAMP was specific and recognized DNA equally well from other *T. b. rhodesiense* strains (LouTat 1A, GYBO, IL1501), but did not recognize DNA from *T. b. gambiense* isolates (IL 3258, DAL 972, DAL 072, DAL 069, IPR SG-1020, FONT l′93, JUA, MOS, MA 002) (not shown). It also recognized *T. b. brucei* strain 927 genomic DNA at very high concentrations (i.e. >1 ng DNA/µL equivalent to >10^4^ parasites/µL), but it was specific for *T. b. rhodesiense* at the concentrations tested (10 pg to 0.1 fg DNA/µL equivalent to 10^2^ to 10^−3^ parasites/µL). Negative controls included the eukaryotic protozoan parasites *Babesia microti*, *Plasmodium falciparum*, *Plasmodium ovale*, and *Toxoplasma gondii*, as well as DNA from clinical samples or spiked blood samples, such as *Borrelia burgdorferi*, *Borrelia crocidurae*, *Enterococcus* spp., *Ehrlichia chaffeensis*, *Escherichia coli*, *Pseudomonas aeruginosa*, *Rickettsia parkeri*, *Staphylococcus* spp., and DNA from mouse and human blood. Furthermore, using PSEUDO-*SRA* LAMP under carefully controlled conditions, no false positives were found when DNA from 192 normal human CSF samples was tested. Although it is possible to detect very low parasite numbers using Psuedo-SRA LAMP, the assay's sensitivity is a potential drawback because of risk for amplicon contamination. Therefore, until more validation is done, we do not propose PSEUDO-*SRA* LAMP for diagnosis of *T. b. rhodesiense.* However, the range of sensitivity made it an ideal choice to study the effects of detergent on increasing the ability of LAMP to detect live parasites in biological samples.

### LAMP in human CSF spiked with parasites

**Figure 2:**
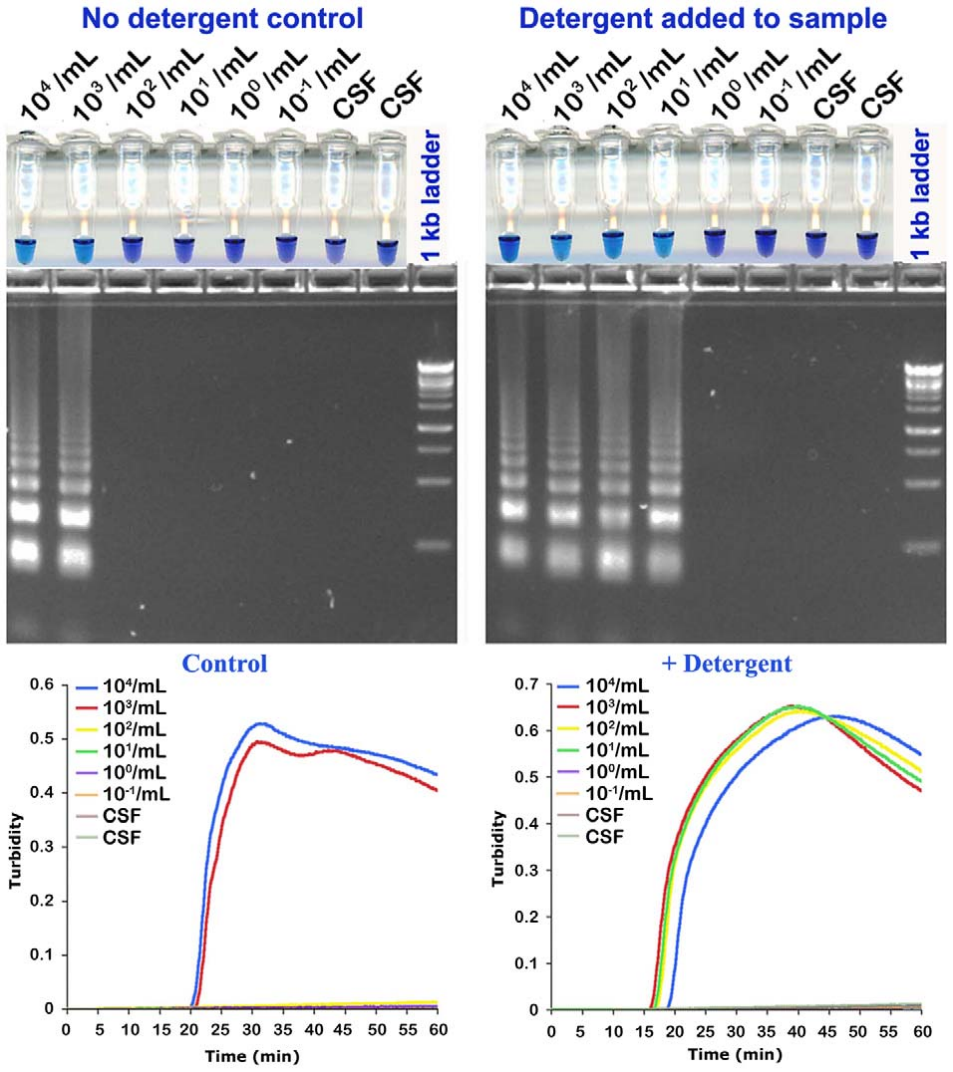
Detergent increases analytical sensitivity of RIME LAMP for the direct detection of *T. b. rhodesiense* in human CSF. Fifty µL water (DNAse/RNAse free) or 10% Triton X-100 was added to human CSF. *T. b. rhodesiense* IL1852 was spiked into 950 µL human CSF without and with 0.5% Triton X-100. The samples were serially diluted in duplicate in normal or detergent treated CSF and incubated at ambient temperature for 60 min. One µL aliquots were assayed for 1 hr at 62°C using RIME LAMP primers. Normal CSF (1 µL) with or without Triton X-100 was used as a control. Each panel shows hydroxynaphthol blue reaction tubes (**top**), agarose gel (**center**) and real-time turbidity data (**bottom**) from the same samples.

**Figure 3:**
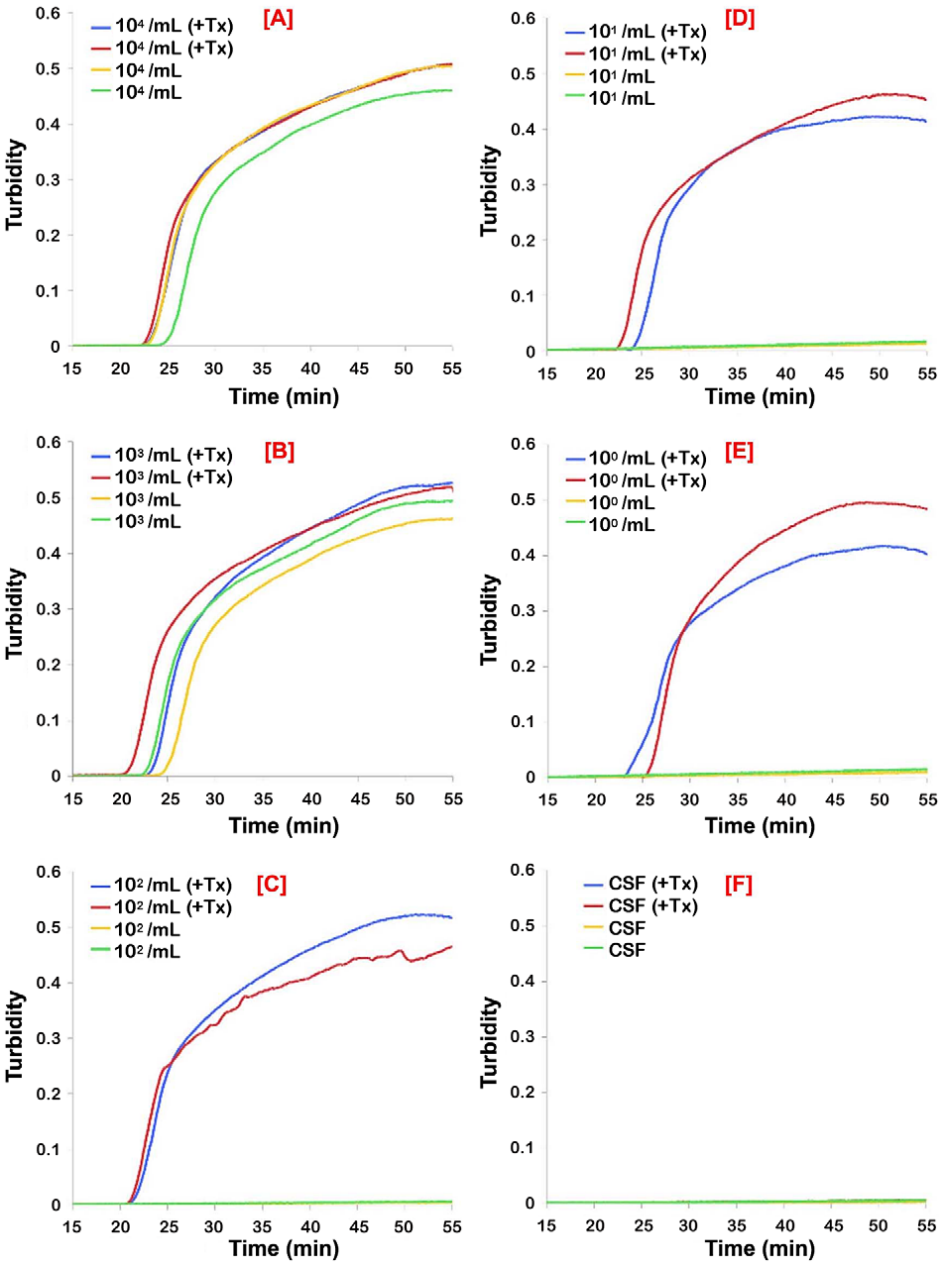
Analytical sensitivity of real-time PSEUDO-*SRA* LAMP for the direct detection of *T. b. rhodesiense* in human CSF. *T. b. rhodesiense* IL1852 was spiked into human CSF without and with 0.5% Triton X-100 (+Tx). The samples were serially diluted in normal or detergent treated CSF. After 60 min incubation at ambient temperature, 1 µL aliquots were assayed using the PSEUDO-*SRA* LAMP primers. Normal CSF with or without Triton X-100 was used as a control. The data for each individual sample is presented as real-time turbidity values versus LAMP reaction time. The number of parasites/mL CSF originally present in the sample used for the assays in the panels shown are: [**A**], 10^4^/mL; [**B**], 10^3^/mL; [**C**], 10^2^/mL; [**D**], 10^1^/mL; [**E**], 10^0^/mL; [**F**], CSF alone.

To mimic a clinical situation, we first tested RIME LAMP and PSEUDO-*SRA* LAMP on human CSF spiked with live *T. b. rhodesiense* IL1852 and analyzed the reaction products on agarose gels, HNB reaction, and/or real-time LAMP based on turbidimetric readings. As predicted, the LAMP assays had a detection limit of 10^3^ parasites per mL based on 1 µL assay samples for RIME (Fig. 2) and PSEUDO-*SRA* LAMP (Fig. 3). While sensitivity could be increased up to 10 fold by increasing CSF sample volume to 10 µL (not shown), the presence of detergent (i.e. 0.5% Triton X-100) alone added to the CSF samples improved detection to 10 and 1 parasite/mL, representing a 100 to 1000-fold increase in RIME LAMP and PSEUDO-*SRA* LAMP assay analytical sensitivity, respectively (Figs 2 and 3; Table 1). Release of parasite DNA by 0.5% Triton required between 30 and 60 min incubation.

**Table 1:** Summary of LAMP assays conducted using trypanosome spiked human CSF and blood.

LAMP assay conditions					Trypanosomes/mL							
Source of DNA	How sample assayed	Triton added to		Primer set	10^4^	10^3^	10^2^	10^1^	10^0^	10^−1^	10^−2^	None
		Sample	Card									
CSF	Direct	No	N/A	RIME	**+**	**+**	**−**	**−**	**−**	**−**	nd	−
		Yes	N/A	RIME	**+**	**+**	**+**	**+**	**−**	**−**	nd	−
		No	N/A	PSEUDO-SRA	**+**	**+**	**–**	**–**	**–**	**–**	nd	−
		Yes	N/A	PSEUDO-SRA	**+**	**+**	**+**	**+**	**+**	**+/–**	nd	−
Blood	DBS	No	No	RIME	**+**	**+**	**−**	**−**	**−**	**−**	nd	−
		No	Yes	RIME	**+**	**+**	**+**	**+**	**−**	**−**	nd	−
		No	No	PSEUDO-SRA	nd	**+**	**+/−**	**−**	**−**	**−**	**−**	−
		No	Yes	PSEUDO-SRA	nd	**+**	**+**	**+**	**+**	**+**	**+**	−
Blood	DBS	No	No	RIME	**+**	**+**	**−**	**−**	**−**	**−**	nd	−
		Yes	No	RIME	**+**	**+**	**+**	**+**	**−**	**−**	nd	−
		No	No	PSEUDO-SRA	**+**	**+**	**+**	**+**	**−**	**−**	nd	−
		Yes	No	PSEUDO-SRA	**+**	**+**	**+**	**+**	**+**	**+/−**	nd	−

**+** **= ** All replicates positive; **+/–** **= ** positive/negative mix; **−  = ** All replicates negative; nd ** = ** not done; N/A ** = ** not applicable; DBS ** = ** dried blood spot on 903 card.

### LAMP assay using dried blood spots (DBS) of human blood spiked with parasites

The transport and storage of DBS or CSF on filter paper cards is a common practice in the field. DBS on Whatman Protein Saver 903 cards are used for parasite pathogen detection (DNA, RNA and/or protein) and genotyping [38], [39], [40], [41], [42], [43]. Depending on the paper matrix, DNA, RNA and/or protein to be tested are first extracted from defined diameter punches (e.g. 2 mm) and 1–5 uL are assayed. Assay sensitivity for trypanosomes is limited by the stoichiometric presence of the parasite in the assayed sample. Analytical sensitivity is further reduced since sample volumes in filter paper punches represent <1% of the total captured on the paper itself [44].

**Figure 4:**
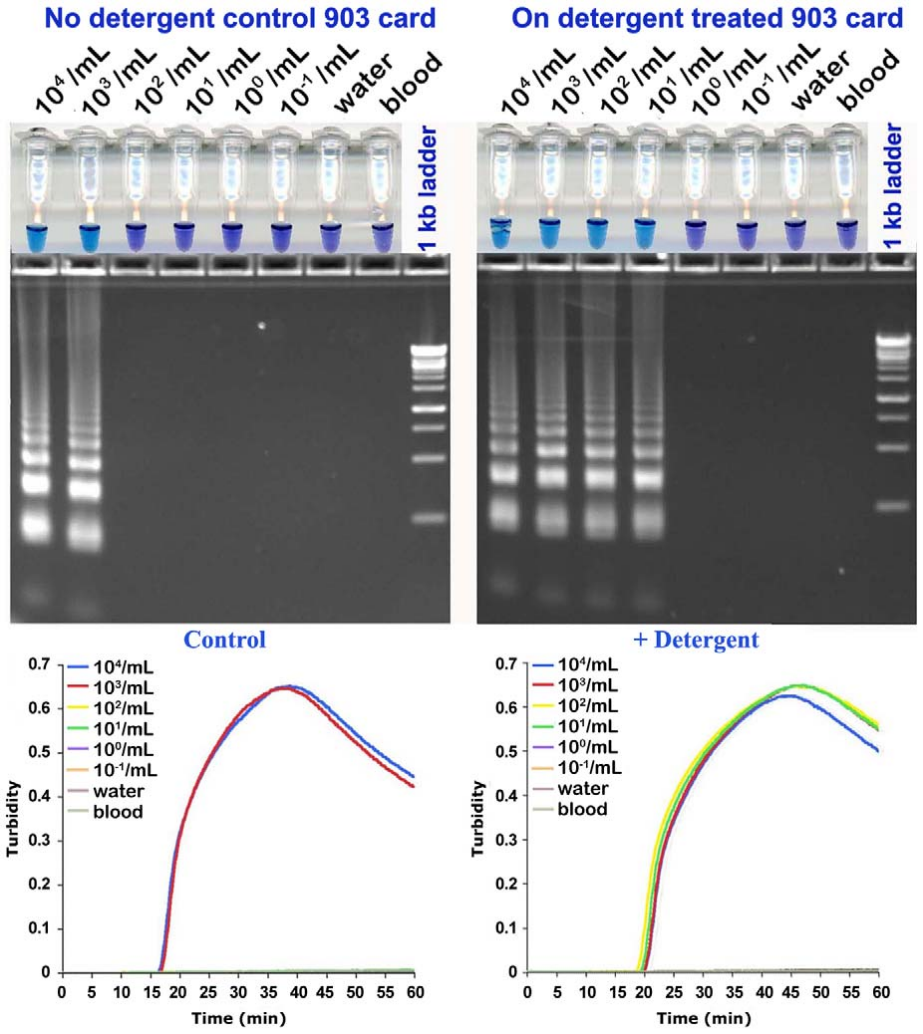
Analytical sensitivity of RIME LAMP for detection of *T. b. rhodesiense* DNA in human blood. *T. b. rhodesiense* IL1852 was spiked into whole human blood, serially diluted and spotted in duplicate on Protein Saver 903 cards or 903 cards pretreated with 0.5% Triton X-100 and allowed to dry overnight. DNA from the DBS was extracted as described in the methods [37] and 1 µL aliquots assayed using RIME LAMP primers. Normal blood (blood) or nuclease free water with or without Triton X-100 were used as controls. Each panel shows hydroxynaphthol blue reaction tubes (**top**), agarose gel (**center**) and real-time turbidity data (**bottom**) from the same samples. DBS DNA from uninfected blood was used as a negative control.

**Figure 5:**
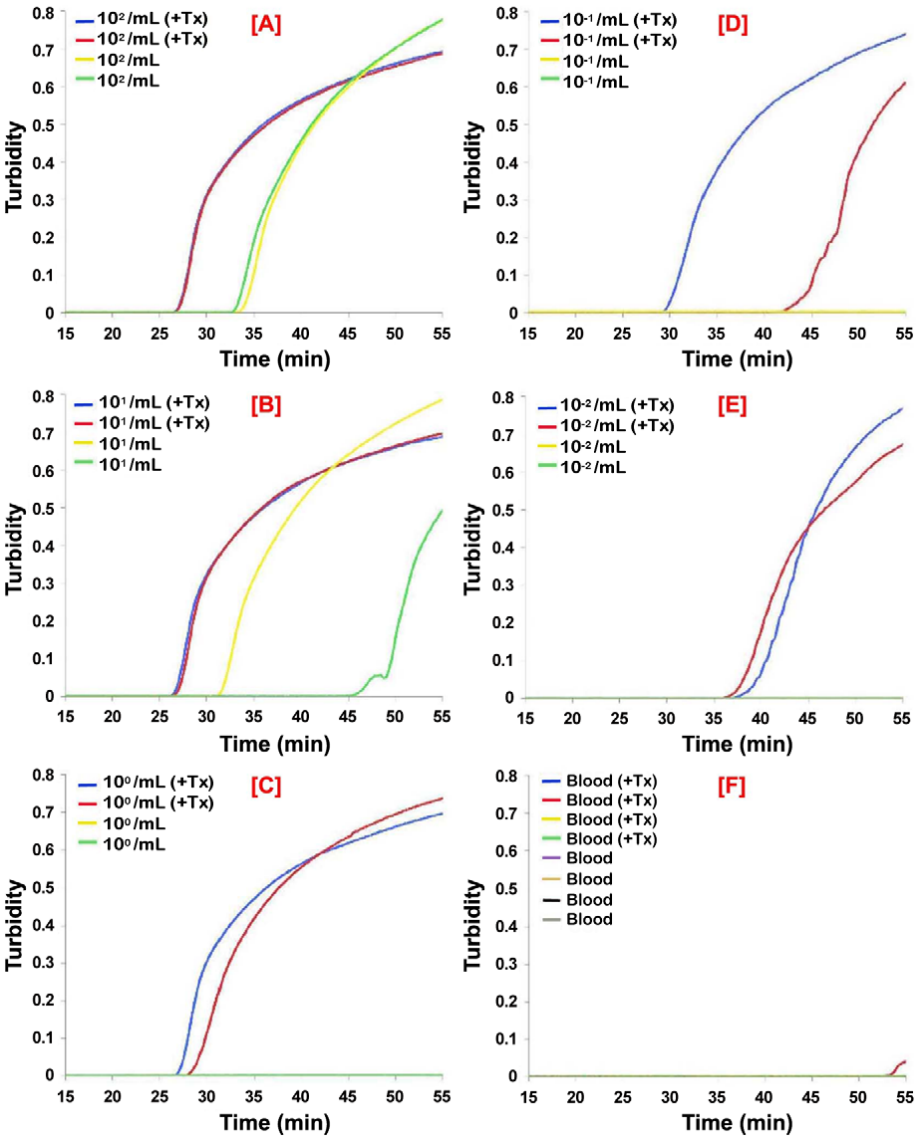
Analytical sensitivity of PSEUDO-*SRA* LAMP for detection of *T. b. rhodesiense* DNA in human blood. *T. b. rhodesiense* IL1852 was spiked into whole human blood, serially diluted and spotted in duplicate on Protein Saver 903 cards or 903 cards pretreated with 0.5% Triton X-100 (+ Tx) and allowed to dry overnight. Control blood samples without trypanosomes were spotted in quadruplicate. DNA from individual DBS (n = 2) was extracted as described in the methods and 1 µL aliquots assayed using PSEUDO-*SRA* LAMP primers. DBS DNA from uninfected blood was used as a negative control (n = 4 +/− Triton). The number of parasites/mL blood originally present in the sample used for the assays in the panels shown are: [**A**], 10^2^/mL; [**B**], 10^1^/mL; [**C**], 10^0^/mL; [**D**], 10^−1^/mL; [**E**], 10^−2^/mL; [**F**], blood alone.

We used parasite-spiked human blood spotted on dry Protein Saver 903 cards pretreated with detergent. Remarkably, the presence of detergent greatly enhanced LAMP detection limits for parasite DNA by about 100 fold for RIME and PSEUDO-*SRA* LAMP (Figs 4 and 5; Table 1). Enhanced detection sensitivity was also found when *T. b. rhodesiense* IL1852 DNA was extracted from DBS from Protein Saver 903 cards containing normal or detergent-treated parasite-spiked human blood (Figs S2 and S3). In general, replicates were more reproducible in assays where the detergent was present in the paper. The presence of detergent had no effect on analytical sensitivity by HNB [26], confirming its use for easy, inexpensive, accurate, and reliable field detection of LAMP-amplified DNA. As with any DNA amplification method, standard precautions for avoiding template contamination [45] also apply for LAMP-based assays.

### Added Advantages

It has been shown that sensitivity, including detection of type 1 *T. b. gambiense* [20], can be greatly enhanced after heat denaturing the samples before LAMP assay [16], [17], [20]. However, this procedure is less convenient than simply incubating samples at ambient temperature with detergent or allowing samples to dry on detergent-pretreated filter cards. Aerosol effects by heating the samples could also increase the risk of cross-contamination prior to addition of the reaction mixture. Furthermore, the extra steps required for techniques such as quantitative buffy coat, microhematocrit centrifugation (mHCT), mini-anion-exchange centrifugation technique (mAECT) used to concentrate the parasites from blood or CSF [6], [7] also increase contamination risk. The addition of samples directly in the reaction helps reduce contamination.

Recent findings by Deborggraeve *et al.* [46] suggest that while PCR performed better than, or similar to current parasite detection techniques for *T. b. gambiense* sleeping sickness diagnosis and staging, it cannot be used for post-treatment follow-up because of persistence of living or dead parasites or their DNA after successful treatment. The use of LAMP on serially diluted sample in the absence and presence of detergent could be useful for differentiating between these scenarios; large difference might indicate a recent infection with small differences indicating persistent or relapse infection. While we have not yet optimized conditions with regards to detergent concentration or class (nonionic, ionic or zwitterionic), our preliminary evidence supports the concept that a detergent such as Triton X-100 can be used in a variety of ways to enhance the analytical sensitivity of multi-copy gene LAMP-based assays for the detection of intact African trypanosomes in blood and CSF approximately approaching or reaching the detection limits of LAMP for genomic DNA.

### Conclusion

In addition to LAMP, the implications of these findings are far reaching and should also be applicable for improved lateral-flow dipstick methods recently introduced [47], PCR, or other nucleic acid amplification-based [Recombinase Polymerase Amplification (TwistDX), Strand Displacement Amplification (Probetec ET, Becton-Dickinson), Nucleic Acid Sequenced Base Amplification (Primer Biosoft International)] technologies where microbial pathogen, including protozoan parasite (e.g. *Plasmodium*) DNA/RNA could be easily released by detergents. Unlocking the potential power of LAMP for accurate HAT diagnosis presents an excellent option for the administration of effective anti-trypanosome treatment. In summary, the procedure paves the way for the adaptation of LAMP and similar technologies as simple cost-effective diagnostics for intact African trypanosomes in humans, animals and tsetse flies, and also for other protozoan parasites and microorganisms that cause diseases that plague the developing world.

## Supporting Information

Figure S1 ***T. b. rhodesiense*** **IL1852 contains the** ***SRA*** **gene.** Genomic DNA isolated from IL1852 trypanosomes was checked by PCR using oligonucleotide primers directed against the *SRA* gene diagnostic for *T. b. rhodesiense*, and the *TgsGP* gene diagnostic for *T. b. gambiense*. Positive controls included in each reaction were ELIANE, a *T. b. gambiense* group 1 from Côte d'Ivoire [49], and Z222, a confirmed *T. b. rhodesiense* from Zambia.
(TIF)

Figure S2 **Analytical sensitivity of RIME LAMP for dried blood spot detection of** ***T. b. rhodesiense*** **DNA from detergent treated human blood spotted on 903 cards.** Fifty µL water (DNAse/RNAse free) or 10% Triton X-100 was added to 950 µL human blood. *T. b. rhodesiense* IL1852 was spiked into human blood without and with 0.5% (w/v) Triton X-100. The samples were serially diluted in normal or detergent treated blood and spotted on Protein Saver 903 cards. DNA from the DBS was extracted [37] and 1 µL aliquots assayed using RIME LAMP primers. Each panel shows hydroxynaphthol blue reaction tubes (**top**), agarose gel (**center**) and real-time turbidity data (**bottom**) from the same samples. DBS DNA from uninfected blood was used as a negative control.
(TIF)

Figure S3 **Analytical sensitivity of PSEUDO-** ***SRA*** **for dried blood spot detection of** ***T. b. rhodesiense*** **DNA from detergent treated human blood spotted on 903 cards.** *T. b. rhodesiense* IL1852 was spiked and serially diluted into human blood without and with 0.5% Triton X-100 (+Tx) and spotted on paper cards as in Fig. 4. The DNA from the DBS was extracted and 1 µL aliquots assayed using PSEUDO-*SRA* LAMP primers. The data for each individual sample is presented as real-time turbidity values versus LAMP reaction time. DBS DNA from uninfected blood was used as a negative control. The number of parasites/mL blood in the panels shown are: [**A**], 10^3^/mL; [**B**], 10^2^/mL; [**C**], 10^1^/mL; [**D**], 10^0^/mL; [**E**], 10^−1^/mL; [**F**], blood alone.
(TIF)

Table S1 ***SRA*** **gene 5′-3′ sequence targeted by PSEUDO-** ***SRA*** **(AJ560644).**
(DOC)

## References

[1] Docampo R, Moreno SN (2003). Current chemotherapy of human African trypanosomiasis.. Parasitol Res.

[2] Enanga B, Burchmore RJ, Stewart ML, Barrett MP (2002). Sleeping sickness and the brain.. Cell Mol Life Sci.

[3] Grab DJ, Kennedy PE (2008). Traversal of human and animal trypanosomes across the blood-brain barrier.. J Neurovirology.

[4] Kristensson K, Nygard M, Bertini G, Bentivoglio M (2010). African trypanosome infections of the nervous system: parasite entry and effects on sleep and synaptic functions.. Prog Neurobiol.

[5] Kennedy PG (2004). Human African trypanosomiasis of the CNS: current issues and challenges.. J Clin Invest.

[6] Chappuis F, Loutan L, Simarro P, Lejon V, Büscher P (2005). Options for field diagnosis of human african trypanosomiasis.. Clin Microbiol Rev.

[7] Büscher P, Lejon V (2004).

[8] Koffi M, Solano P, Denizot M, Courtin D, Garcia A (2006). Aparasitemic serological suspects in *Trypanosoma brucei gambiense* human African trypanosomiasis: a potential human reservoir of parasites?. Acta Trop.

[9] Bisser S, Lejon V, Preux PM, Bouteille B, Stanghellini A (2002). Blood-cerebrospinal fluid barrier and intrathecal immunoglobulins compared to field diagnosis of central nervous system involvement in sleeping sickness.. J Neurol Sci.

[10] Solano P, Michel JF, Lefrancois T, de La Rocque S, Sidibe I (1999). Polymerase chain reaction as a diagnosis tool for detecting trypanosomes in naturally infected cattle in Burkina Faso.. Vet Parasitol.

[11] Kuboki N, Inoue N, Sakurai T, Di Cello F, Grab DJ (2003). Loop-mediated isothermal amplification for detection of African trypanosomes.. J Clin Microbiol.

[12] Mori Y, Notomi T (2009). Loop-mediated isothermal amplification (LAMP): a rapid, accurate, and cost-effective diagnostic method for infectious diseases.. J Infect Chemother.

[13] Thekisoe OM, Bazie RS, Coronel-Servian AM, Sugimoto C, Kawazu S (2009). Stability of Loop-Mediated Isothermal Amplification (LAMP) reagents and its amplification efficiency on crude trypanosome DNA templates.. J Vet Med Sci.

[14] Thekisoe OM, Kuboki N, Nambota A, Fujisaki K, Sugimoto C (2007). Species-specific loop-mediated isothermal amplification (LAMP) for diagnosis of trypanosomosis.. Acta Trop.

[15] Thekisoe OM, Inoue N, Kuboki N, Tuntasuvan D, Bunnoy W (2005). Evaluation of loop-mediated isothermal amplification (LAMP), PCR and parasitological tests for detection of *Trypanosoma evansi* in experimentally infected pigs.. Vet Parasitol.

[16] Njiru ZK, Mikosza AS, Matovu E, Enyaru JC, Ouma JO (2008). African trypanosomiasis: sensitive and rapid detection of the sub-genus Trypanozoon by loop-mediated isothermal amplification (LAMP) of parasite DNA.. Int J Parasitol.

[17] Njiru ZK, Mikosza AS, Armstrong T, Enyaru JC, Ndung'u JM (2008). Loop-Mediated Isothermal Amplification (LAMP) Method for Rapid Detection of *Trypanosoma brucei rhodesiense*.. PLoS Negl Trop Dis.

[18] Thekisoe OMM, Inoue N, Namangala B, Sugimoto C (2010). Loop-mediated isothermal amplification (LAMP) assays for specific detection of *Trypanosoma vivax* infections in livestock and tsetse flies..

[19] Njiru ZK, Ouma JO, Enyaru JC, Dargantes AP (2010). Loop-mediated Isothermal Amplification (LAMP) test for detection of *Trypanosoma evansi* strain B.. Exp Parasitol.

[20] Njiru ZK, Traub R, Ouma JO, Enyaru JC, Matovu E (2011). Detection of Group 1 *Trypanosoma brucei gambiense* by Loop-mediated isothermal amplification (LAMP).. J Clin Microbiol.

[21] Njiru ZK, Ouma JO, Bateta R, Njeru SE, Ndungu K (2011). Loop-mediated isothermal amplification test for *Trypanosoma vivax* based on satellite repeat DNA.. Vet Parasitol.

[22] Hafner GJ, Yang IC, Wolter LC, Stafford MR, Giffard PM (2001). Isothermal amplification and multimerization of DNA by Bst DNA polymerase..

[23] Nagamine K, Hase T, Notomi T (2002). Accelerated reaction by loop-mediated isothermal amplification using loop primers.. Mol Cell Probes.

[24] Poon LL, Wong BW, Ma EH, Chan KH, Chow LM (2006). Sensitive and inexpensive molecular test for falciparum malaria: detecting *Plasmodium falciparum* DNA directly from heat-treated blood by loop-mediated isothermal amplification.. Clin Chem.

[25] Mori Y, Nagamine K, Tomita N, Notomi T (2001). Detection of loop-mediated isothermal amplification reaction by turbidity derived from magnesium pyrophosphate formation.. Biochem Biophys Res Commun.

[26] Wastling SL, Picozzi K, Kakembo AS, Welburn SC (2010). LAMP for Human African Trypanosomiasis: A Comparative Study of Detection Formats.. PLoS Negl Trop Dis.

[27] Tomita N, Mori Y, Kanda H, Notomi T (2008). Loop-mediated isothermal amplification (LAMP) of gene sequences and simple visual detection of products.. Nat Protoc.

[28] Qiao YM, Guo YC, Zhang XE, Zhou YF, Zhang ZP (2007). Loop-mediated isothermal amplification for rapid detection of *Bacillus anthracis* spores.. Biotechnol Lett.

[29] Matovu E, Kuepfer I, Boobo A, Kibona S, Burri C (2010). Comparative detection of trypanosomal DNA by loop-mediated isothermal amplification and PCR from flinders technology associates cards spotted with patient blood.. J Clin Microbiol.

[30] Notomi T, Okayama H, Masubuchi H, Yonekawa T, Watanabe K (2000). Loop-mediated isothermal amplification of DNA.. Nucleic Acids Res.

[31] Mhando PJ, Yanagi T, Fukuma T, Nakazawa S, Kanaba H (1987). *In vitro* cultivation of *Trypanosoma brucei* subspecies with cells derived from brain and mouse of new born mouse.. Trop Med.

[32] Eshita Y, Urakawa T, Hirumi H, Fish WR, Majiwa PA (1992). Metacyclic form-specific variable surface glycoprotein-encoding genes of *Trypanosoma (Nannomonas) congolense*.. Gene.

[33] Nikolskaia OV, de ALAP, Kim YV, Lonsdale-Eccles JD, Fukuma T (2008). Erratum.. J Clin Invest.

[34] Radwanska M, Chamekh M, Vanhamme L, Claes F, Magez S (2002). The serum resistance-associated gene as a diagnostic tool for the detection of *Trypanosoma brucei rhodesiense*.. Am J Trop Med Hyg.

[35] Radwanska M, Claes F, Magez S, Magnus E, Perez-Morga D (2002). Novel primer sequences for polymerase chain reaction-based detection of *Trypanosoma brucei gambiense*.. Am J Trop Med Hyg.

[36] Pays E, Dekerck P, Van Assel S, Babiker EA, Le Ray D (1983). Comparative analysis of a *Trypanosoma brucei gambiense* antigen gene family and its potential use in epidemiology of sleeping sickness.. Mol Biochem Parasitol.

[37] Sambrook J, Russell DW, Sambrook J, Russell DW (2001). Preparation and analysis of eukaryotic genomic DNA;.

[38] Stresman GH, Kamanga A, Moono P, Hamapumbu H, Mharakurwa S (2010). A method of active case detection to target reservoirs of asymptomatic malaria and gametocyte carriers in a rural area in Southern Province, Zambia.. Malar J.

[39] Mlambo G, Vasquez Y, LeBlanc R, Sullivan D, Kumar N (2008). A filter paper method for the detection of *Plasmodium falciparum* gametocytes by reverse transcription polymerase chain reaction.. Am J Trop Med Hyg.

[40] Buckton AJ (2008). New methods for the surveillance of HIV drug resistance in the resource poor world.. Curr Opin Infect Dis.

[41] Johannessen A, Garrido C, Zahonero N, Sandvik L, Naman E (2009). Dried blood spots perform well in viral load monitoring of patients who receive antiretroviral treatment in rural Tanzania.. Clin Infect Dis.

[42] Garrido C, Zahonero N, Corral A, Arredondo M, Soriano V (2009). Correlation between human immunodeficiency virus type 1 (HIV-1) RNA measurements obtained with dried blood spots and those obtained with plasma by use of Nuclisens EasyQ HIV-1 and Abbott RealTime HIV load tests.. J Clin Microbiol.

[43] Recommended genotyping procedures (RGPs) to identify parasite populations; 2007 May 29-31; Amsterdam, The Netherlands

[44] Cox AP, Tosas O, Tilley A, Picozzi K, Coleman P (2010). Constraints to estimating the prevalence of trypanosome infections in East African zebu cattle.. Parasit Vectors.

[45] Hildebrandt F, Singh-Sawhney I, Hildebrandt F, Igarashi P (1999). Polymerase Chain Reaction.. Techniques in molecular medicine: Springer-Verlag.

[46] Deborggraeve S, Lejon V, Ekangu RA, Mumba Ngoyi D, Pati Pyana P (2011). Diagnostic Accuracy of PCR in gambiense Sleeping Sickness Diagnosis, Staging and Post-Treatment Follow-Up: A 2-year Longitudinal Study.. PLoS Negl Trop Dis.

[47] Njiru ZK (2011). Rapid and sensitive detection of human African trypanosomiasis by loop-mediated isothermal amplification combined with a lateral-flow dipstick.. Diagn Microbiol Infect Dis.

[48] Inverso JA, Uphoff TS, Johnson SC, Paulnock DM, Mansfield JM (2010). Biological variation among african trypanosomes: I. Clonal expression of virulence is not linked to the variant surface glycoprotein or the variant surface glycoprotein gene telomeric expression site.. DNA Cell Biol.

[49] Felgner P, Brinkmann U, Zillmann U, Mehlitz D, Abu-Ishira S (1981). Epidemiological studies on the animal reservoir of gambiense sleeping sickness. II. Parasitolgical and immunodiagnostic examination of the human population.. Tropenmed Parasitol.

