# Supplementary figures and images for: Using Detergent to Enhance Detection Sensitivity of African Trypanosomes in Human CSF and Blood by Loop-Mediated Isothermal Amplification (LAMP)
Source: PLoS Negl Trop Dis. 2011 Aug 2;5(8):e1249. doi: 10.1371/journal.pntd.0001249 (PMC3149022; doi:10.1371/journal.pntd.0001249)

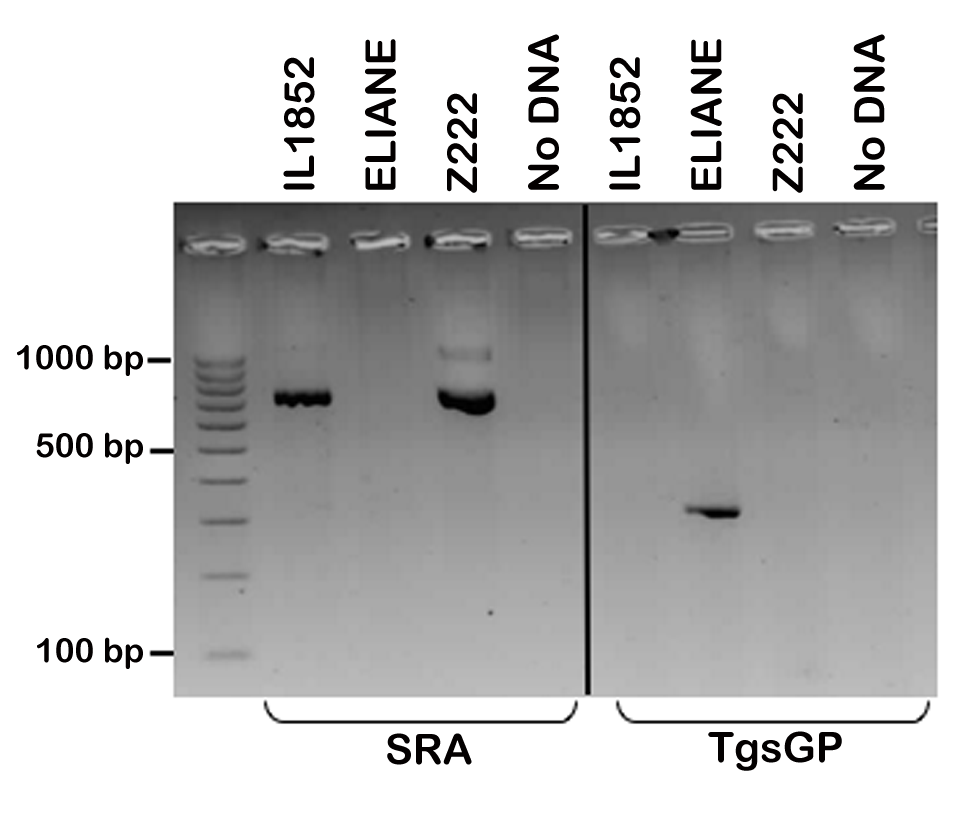

Supplement: Figure S1 — T. b. rhodesiense IL1852 contains the SRA gene. Genomic DNA isolated from IL1852 trypanosomes was checked by PCR using oligonucleotide primers directed against the SRA gene diagnostic for T. b. rhodesiense, and the TgsGP gene diagnostic for T. b. gambiense. Positive controls included in each reaction were ELIANE, a T. b. gambiense group 1 from Côte d'Ivoire [49], and Z222, a confirmed T. b. rhodesiense from Zambia. (TIF) [file pntd.0001249.s001.tif]

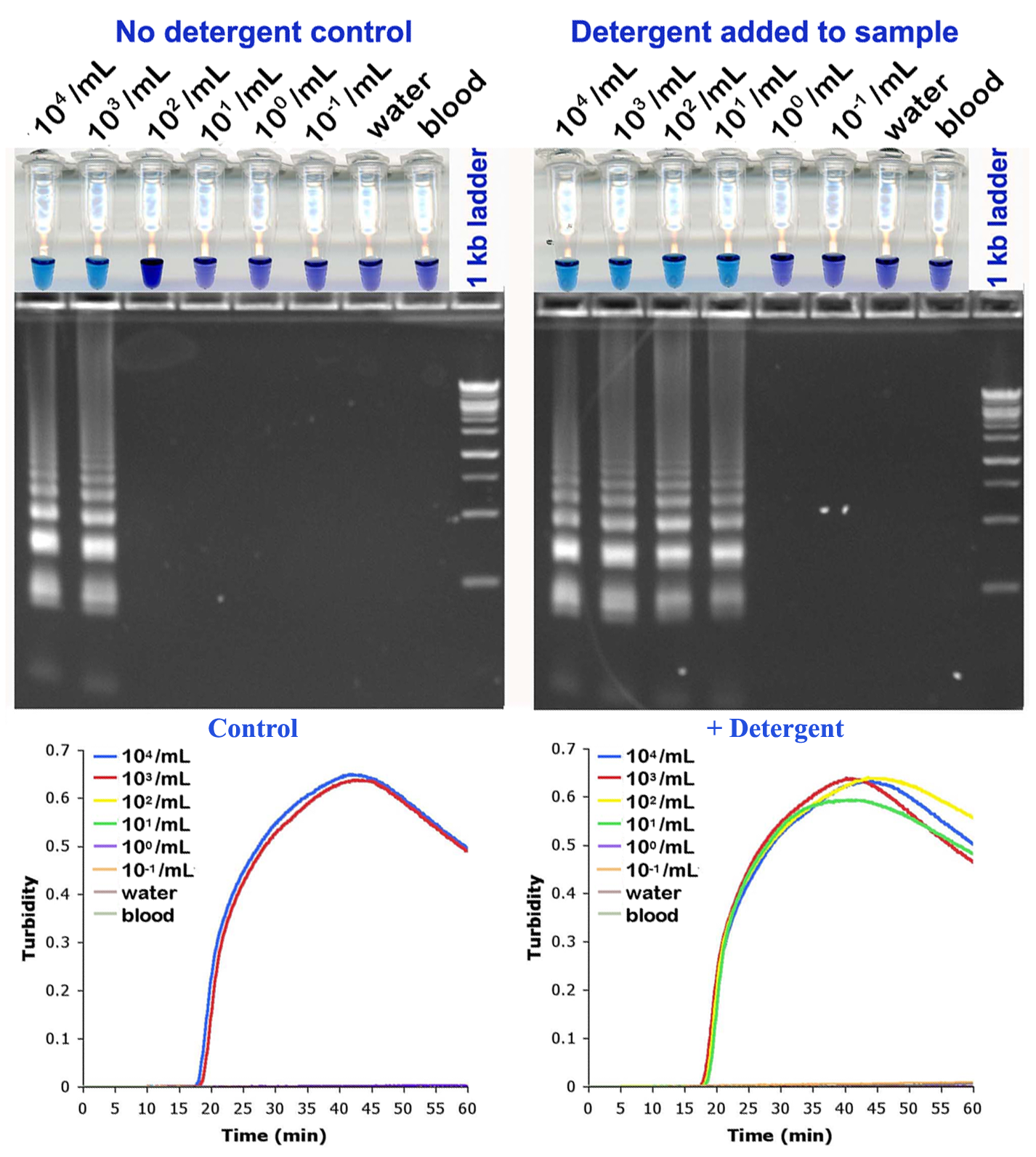

Supplement: Figure S2 — Analytical sensitivity of RIME LAMP for dried blood spot detection of T. b. rhodesiense DNA from detergent treated human blood spotted on 903 cards. Fifty µL water (DNAse/RNAse free) or 10% Triton X-100 was added to 950 µL human blood. T. b. rhodesiense IL1852 was spiked into human blood without and with 0.5% (w/v) Triton X-100. The samples were serially diluted in normal or detergent treated blood and spotted on Protein Saver 903 cards. DNA from the DBS was extracted [37] and 1 µL aliquots assayed using RIME LAMP primers. Each panel shows hydroxynaphthol blue reaction tubes (top), agarose gel (center) and real-time turbidity data (bottom) from the same samples. DBS DNA from uninfected blood was used as a negative control. (TIF) [file pntd.0001249.s002.tif]

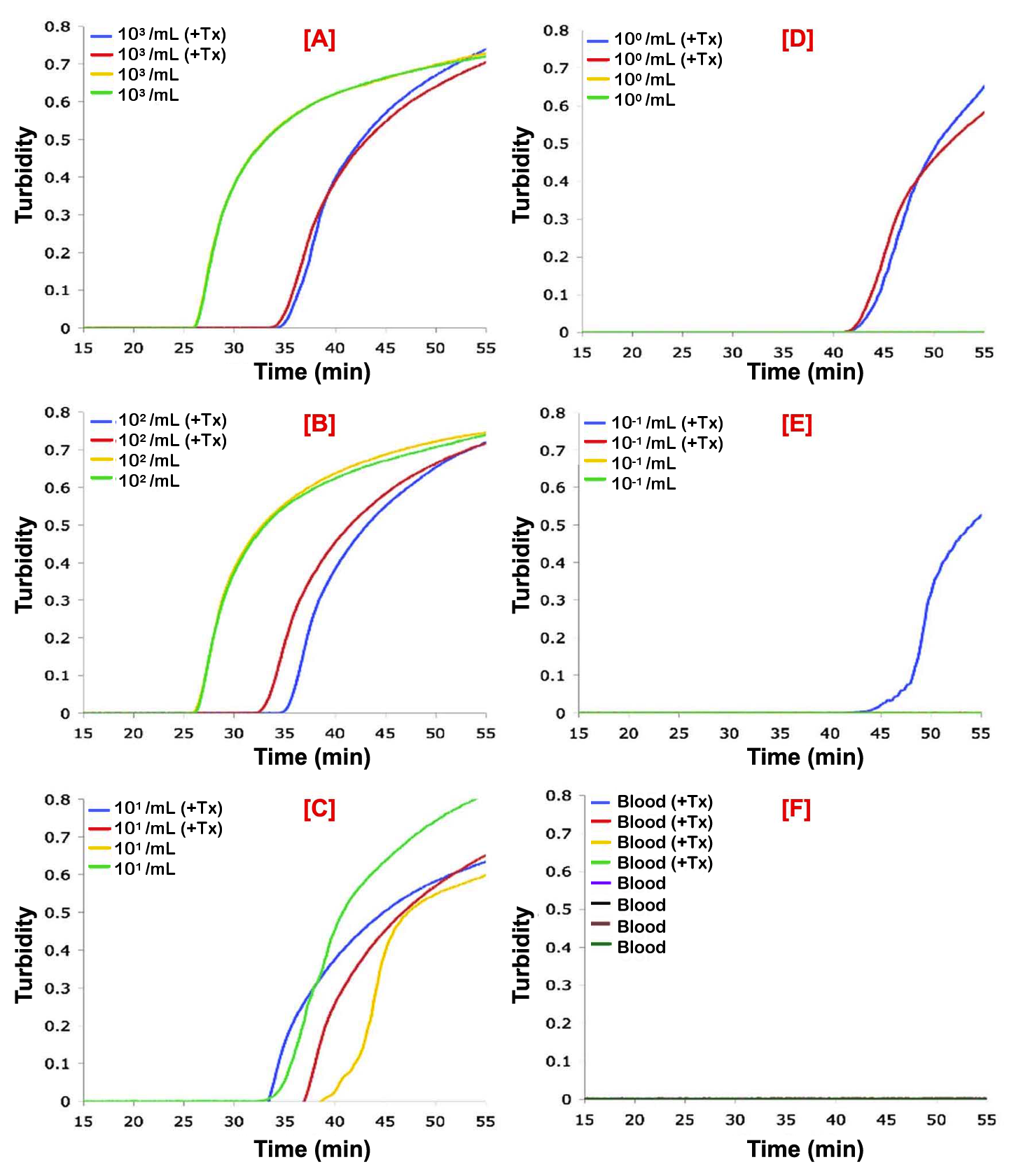

Supplement: Figure S3 — Analytical sensitivity of PSEUDO- SRA for dried blood spot detection of T. b. rhodesiense DNA from detergent treated human blood spotted on 903 cards. T. b. rhodesiense IL1852 was spiked and serially diluted into human blood without and with 0.5% Triton X-100 (+Tx) and spotted on paper cards as in Fig. 4. The DNA from the DBS was extracted and 1 µL aliquots assayed using PSEUDO-SRA LAMP primers. The data for each individual sample is presented as real-time turbidity values versus LAMP reaction time. DBS DNA from uninfected blood was used as a negative control. The number of parasites/mL blood in the panels shown are: [A], 103/mL; [B], 102/mL; [C], 101/mL; [D], 100/mL; [E], 10−1/mL; [F], blood alone. (TIF) [file pntd.0001249.s003.tif]
