# Supplementary material for: Using Detergent to Enhance Detection Sensitivity of African Trypanosomes in Human CSF and Blood by Loop-Mediated Isothermal Amplification (LAMP)
Source: PLoS Negl Trop Dis. 2011 Aug 2;5(8):e1249. doi: 10.1371/journal.pntd.0001249 (PMC3149022; doi:10.1371/journal.pntd.0001249)
Supplement: Table S1 — SRA gene 5′-3′ sequence targeted by PSEUDO- SRA (AJ560644). (DOC) [file pntd.0001249.s004.doc]

| **Table S1**. *SRA* gene 5’-3’ sequence targeted by PSEUDO-*SRA* (AJ560644) |
| --- |
| TACTACCTCTTTCACACTATGTTTATAGCGTTTAAACGATATGTGTGTGCGGAGTGGTGGTGGACTAAAG  CAACATATACATTGACAGGCGAGAGCAGAATCTACAAACTCAAAAGCAAAAAAATGGCGAGTAAACAGGA  AGCTGTCACTGTATAAACACAACTGCGGATTTATTTTTTCTCCTAGAAGACACACCTCTAAGAATCACAA  TAGCAAGGCGAACCGAAAGCGAAGCACAGTTAACGTAACAGCAATGCCCCGAAATTCGGGCCGGACAACA  AGTACCTTGGCGCTCGCGCTGGCCCTAAAGCTGCTGGCAGTGCCTGTATCGCCCAGTGGCGCCGCCTTTG  ACGAAGAGCCCGTCAAGAAGGTTTGCAAAGTAGAAAAAAACTTAGCAGACGTCGCAGGAATCGCTTTGGC  CAAAATAAACAACCTGATAAAAC**AAGTATCGGCAGCAACCG**AAGCGGAAGCAAGAATGACCTTGGCCGCC  GCAAGCACAGACCACAGCAACATCTCAGCGCTTTATGCCGCGGCGTCAAACATAGTGACAAGATGCGTAC  TCAACGCAGTCCACGCTCTTACAAGTCTTGCGCCAATAGCGTTAACTGCAG**CGACCAACGGAGCCAAAA**C  CAGTGGGCACATCTCAGAAGTAATCGACATTCTGCAGCAGGCGTCACAAGGTAAGACAGAAGGAAAGTGC  ATAGTGAAAAGCGGCGGCGGTACAACAACAGTA |
| **Legend:** Sites targeted by PSEUDO-*SRA* LAMP based on **F3** (5’-**AAGTATCGGCAGCAACCG**-3’) and  strand complementary to **B3** (5’-**CGACCAACGGAGCCAAAA**-3’) |
